# From Network Governance to Real-World-Time Learning: A High-Reliability Operating Model for Rare Cancers

**DOI:** 10.3390/cancers18040643

**Published:** 2026-02-16

**Authors:** Bruno Fuchs, Anna L. Falkowski, Ruben Jaeger, Barbara Kopf, Christian Rothermundt, Kim van Oudenaarde, Ralph Zacchariah, Philip Heesen, Georg Schelling, Gabriela Studer

**Affiliations:** 1 Faculty of Health Sciences & Medicine, University of Lucerne, Frohburgstrasse 3, 6002 Luzern, Switzerland; 2 Swiss Sarcoma Network SSN, LUKS Sarcoma-IPU, University Teaching Hospital LUKS, Spitalstrasse, 6000 Luzern, Switzerland; 3 Department of Orthopaedics and Trauma, LUKS Sarcoma-IPU, University Teaching Hospital LUKS, Spitalstrasse, 6000 Lucerne, Switzerland; 4 Department of Orthopaedics and Trauma, Kantonsspital Winterthur, KSW Sarcoma Center, Brauerstrasse 15, 8401 Winterthur, Switzerland; 5 Istituto Oncologico della Svizzera Italiana, Ente Ospedaliero Cantonale, Ospedale Regionale di Locarno, La Carità, Viale Officina 3, 6500 Bellinzona, Switzerland; 6 Department of Medical Oncology, LUKS Sarcoma-IPU, University Teaching Hospital LUKS, Spitalstrasse, 6000 Lucerne, Switzerland; 7 Department of Radiology, LUKS Sarcoma-IPU, University Teaching Hospital LUKS, Spitalstrasse, 6000 Lucerne, Switzerland; 8 Department of Medical Oncology, Kantonsspital Winterthur, KSW Sarcoma Center, Brauerstrasse 15, 8401 Winterthur, Switzerland; 9 Faculty of Medicine, University of Zurich, Raemistrasse 71, 8006 Zurich, Switzerland; 10 Department of Radiation Oncology, LUKS Sarcoma-IPU, University Teaching Hospital LUKS, Spitalstrasse, 6000 Lucerne, Switzerland

**Keywords:** learning health system, rare cancers, sarcoma, hub-and-spoke, multidisciplinary tumor board/MDT, value-based healthcare, real-world-time data

## Abstract

Background: Rare cancers combine low incidence with high biological heterogeneity and multi-institutional care trajectories. These features make single-center learning structurally incomplete and render pathway fragmentation a dominant driver of preventable harm, variability, and waste. In this context, care quality is best understood as a property of pathway integrity across routing, diagnostics (imaging/biopsy planning), multidisciplinary intent-setting, definitive treatment, and surveillance—rather than as a department-level attribute. Objective: To define a pragmatic, transferable operating blueprint for a rare-cancer Learning Health System (LHS) that turns routine care into continuous, auditable learning under explicit governance, while maintaining claims discipline and protecting measurement validity. Approach: We synthesize an implementation-oriented operating model using the Swiss Sarcoma Network (SSN) as an exemplar. The blueprint couples clinical governance (Integrated Practice Unit logic, hub-and-spoke routing, auditable multidisciplinary team decision systems) with an interoperable real-world-time data backbone designed for benchmarking, pathway mapping, and feedback. The operating logic is expressed as a closed-loop control cycle: capture → harmonize → benchmark → learn → implement → re-measure, with explicit owners, minimum requirements, and failure modes. Results/Blueprint: (i) The model specifies a minimal set of data primitives—time-stamped and traceable decision points covering baseline and tumor characteristics, pathway timing, treatment exposure, outcomes and complications, and feasible longitudinal PROMs and PREMs; (ii) a VBHC-ready, multi-domain measurement backbone spanning outcomes, harms, timeliness, function, process fidelity, and resource stewardship; and (iii) two non-negotiable validity guardrails: explicit applicability (“N/A”) rules and mandatory case-mix/complexity stratification. Implementation is treated as a governed step with defined workflow levers, fidelity criteria, balancing measures, and escalation thresholds to prevent “dashboard medicine” and surrogate-driven optimization. Conclusions: This perspective contributes an operating model—not a platform or single intervention—that enables credible improvement science and establishes prerequisites for downstream causal learning and minimum viable digital twins. By distinguishing enabling infrastructure from the governed clinical system as the primary intervention, the blueprint supports scalable, learnable excellence in rare-cancer care while protecting against gaming, inequity, and inference drift. Distinct from generic LHS or VBHC frameworks, this blueprint specifies validity gates required for rare-cancer benchmarking—explicit applicability (“N/A”) rules, denominator integrity/capture completeness disclosure, anti-gaming safeguards, and escalation governance. These elements are critical in rare cancers because small denominators, high heterogeneity, and multi-institutional pathways otherwise make benchmarking prone to artifacts and unsafe inferences.

## Simple Summary

Treating rare cancers is challenging because they are infrequent, biologically diverse, and care pathways are often fragmented as patients move between hospitals for diagnosis and therapy. As a result, avoidable delays, poorly coordinated steps, and inconsistent decision-making can harm patients and also make it hard to learn what works best from routine care. In this article, we propose a practical operating model for running rare-cancer care as a “learning system” that continuously measures its own performance and improves over time. Using sarcoma care in Switzerland as an example, we describe how coordinated multidisciplinary decision-making, reliable patient routing, and a minimal but high-quality data backbone can support fair benchmarking and targeted improvements without relying on a single metric. This blueprint may help networks improve outcomes, reduce avoidable patient burden and inefficiencies, and create better real-world evidence to guide future research, in order to continuously improve patient care.

## 1. Introduction

Rare cancers combine low incidence with high biological heterogeneity and histotype-specific treatment effects, rendering single-center evidence generation structurally incomplete [1]. At the same time, care trajectories are almost inevitably multi-institutional, amplifying the importance of coordination across pathway interfaces—referral, imaging, biopsy planning, multidisciplinary decision-making, definitive treatment, and surveillance. In this context, quality cannot be understood as the result of isolated disciplines or individual decisions [2]. Instead, outcomes are largely shaped by the integrity of the care pathway: whether patients enter the appropriate diagnostic sequence, whether biopsies preserve definitive treatment options, whether multidisciplinary decisions are made early enough to define intent, and whether follow-up is structured to detect clinically meaningful events [3,4].

Sarcoma care illustrates these constraints with particular clarity [5]. Fragmentation in early diagnostic and treatment pathways can trigger a cascade of preventable harm, including delayed imaging, as well as unnecessary follow-up imaging instead of diagnostic work-up, suboptimal biopsy planning, unplanned and incomplete excisions, i.e., compromised margins requiring escalation, and avoidable morbidity and cost [6]. At the same time, it undermines the ability to learn [7]. Data become incomplete, definitions drift across sites, and attribution of outcomes to clinical decisions becomes speculative. The result is a dual failure mode—clinical and epistemic—in which the system produces both avoidable harm and low-quality evidence. This creates a persistent paradox: while stakeholders call for “more evidence,” real-world care pathways repeatedly erode the conditions required to generate credible evidence [8].

Common responses—more guidelines, more expert consensus, or additional randomized trials—are necessary but insufficient [2,9]. Guidelines do not implement themselves, and trials rarely address the pathway-level interventions that dominate rare-cancer performance, such as referral routing, diagnostic workflow design, or cross-site governance. Moreover, rare cancers require continuous learning because the evidence base evolves unevenly across histotypes and modalities, and because real-world variation in pathway execution is itself an important source of outcome variability. What is missing is an explicit operating model with accountable governance, evidence-ready data flows, transparent measurement, and closed-loop feedback that translates observation into improvement [10,11,12].

In this perspective, we define a pragmatic operating model for a rare-cancer Learning Health System (LHS), using the Swiss Sarcoma Network (SSN) as an exemplar [13,14]. The model intentionally couples elements that are often addressed in isolation: clinical governance through auditable multidisciplinary decision systems and full-cycle accountability; network topology that enables hub-and-spoke routing without centralizing all care; an interoperable real-world-time data backbone capable of benchmarking and pathway mapping; and feedback loops that connect measurement to implementation [15,16]. Within this framing, data function as measurement—making variation visible and enabling corrective action [17].

We operationalize the blueprint as a governed closed-loop learning cycle with explicit ownership, minimum requirements, and failure modes at each step [18,19,20,21]. Importantly, the primary intervention is not a digital platform but the governed clinical system itself. Digital infrastructure enables learning, but it does not substitute for decision governance, pathway design, or accountability for outcomes, harms, and costs. By making this distinction explicit, the proposed LHS becomes a testable proposition: pathway integrity can be improved, preventable harm reduced, and patient-centered outcomes and resource use rendered interpretable in the context of complexity and intent.

## 2. The SSN Operating Context: Network, IPU/HASM/MDTB Governance and Learnability

The Swiss Sarcoma Network (SSN) is used as an exemplar because it explicitly links clinical governance, pathway integrity, and learnability [3]. In rare cancers, multi-institutional trajectories are the norm; therefore, outcomes are often driven less by isolated “center effects” than by the reliability of pathway execution across interfaces (routing, diagnostics, multidisciplinary tumor board (MDTB) timing, definitive treatment, and follow-up). SSN’s corpus is unusually coherent in treating quality as an end-to-end system property and in operationalizing learnability rather than assuming it.

Operationally, SSN can be described as a deliberately coupled clinical–digital architecture. In this manuscript, “deliberately coupled” means that governance and data generation are designed as one system: decisions are produced in auditable structures (MDTB intent-setting; IPU accountability; hub-and-spoke routing) and simultaneously rendered measurable through structured, time-stamped capture at those same decision points. The digital backbone is therefore not an adjunct “data lake”, but the instrumentation layer that makes governance observable, comparable across sites, and actionable. Conversely, data infrastructure without governance collapses into retrospective documentation, while governance without structured capture remains non-learnable. Integrated Practice Unit (IPU) logic assigns accountability across the care cycle; a hub-and-spoke model (HASM) operationalizes access and equity via reliable routing; weekly MDTBs function as the auditable decision engine by producing structured, time-stamped decisions on intent and sequencing; and a real-world-time data backbone enables benchmarking, pathway mapping, and feedback [15,16].

This coupling is intentional: none of these elements is sufficient alone. Routing without auditable MDTB governance obscures decision provenance; MDTBs without structured data remain non-learnable; and data infrastructure without governance collapses into retrospective documentation. SSN’s integrated-versus-fragmented pathway work positions fragmentation as an upstream driver of preventable harm—including unplanned resections, margin compromise, and increased local recurrence risk—thereby motivating an operating model that makes pathway performance measurable and improvable, not a single-intervention narrative [6,7].

In this framework, MDTB value is operationalized primarily as decision traceability and reliability rather than assumed clinical “superiority.” Accordingly, MDTB performance is measured with objective governance metrics: (i) MDTB coverage of eligible cases (with explicit eligibility logic), (ii) time-to-MDTB decision from network entry/definitive suspicion, (iii) completeness of structured decision fields (intent, sequencing, key assumptions, and documented rationale for deviations), and (iv) re-discussion or plan-revision rate as a marker of evolving evidence or diagnostic uncertainty. These metrics provide a measurable basis for benchmarking decision governance across sites and for detecting under-coverage, delay, or “paper compliance” without substantive decisions. Where the literature suggests outcome associations for specialized sarcoma boards, these data are treated as contextual support; causal effects within SSN remain subject to the evaluation designs specified in Section 7.

**Figure 1:**
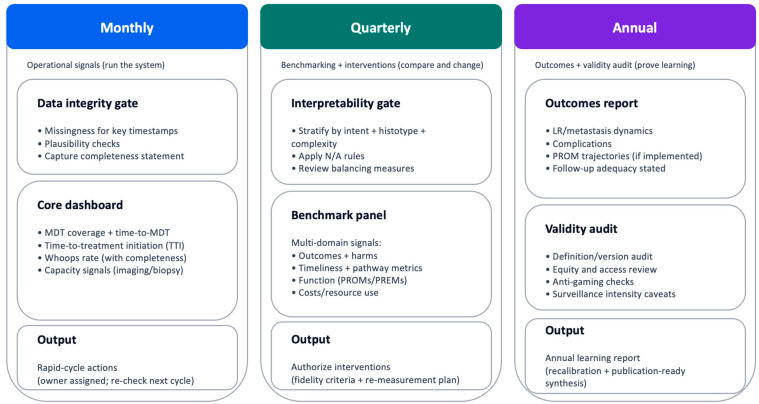
Measurement governance and cadence (minimum viable). Governance cadence that turns indicators into audited change. Monthly reviews (blue) focus on operational signals and data integrity; quarterly cycles (green) support stratified benchmarking, outlier review, and intervention approval with fidelity checks; annual reviews (purple) consolidate outcomes, harms, function/PROMs, costs, and definition/version audits. Escalation triggers (see Box 1) prioritize patient safety and reduce over-interpretation of unstable signals. Abbreviations: LR, local recurrence; MDSG, Measurement and Data Stewardship Group; time-to-treatment initiation; PREMs, patient-reported experience measures; PROMs, patient-reported outcome measures. How to read: Follow the vertical flow to understand the governance cycle; escalation triggers override routine cadence when safety or validity signals deteriorate. N/A, not applicable.

The mechanism is the coupling of (i) team-based intent-setting (MDTB) and accountability across the care cycle (IPU), (ii) reliable routing that determines where expertise must be applied (hub-and-spoke), and (iii) a minimal dataset created at those routed decision points. Together, these elements convert routine multidisciplinary decisions into comparable signals, allowing the network to detect variation, intervene on pathway failures, and re-measure results. Without this coupling, teams remain non-auditable, routing remains opaque, and data remain retrospective. This exemplar anchors the operating loop (Figure 1) and the governance/measurement structure (Figure 1, Box 1, Appendix A).

SSN’s published trajectory provides empirical signals that motivate—rather than “prove”—this operating model. Prior SSN work has linked pathway fragmentation to preventable harm and clinically relevant downstream endpoints (e.g., unplanned resections, margin compromise, and increased local recurrence risk), consistent with the view that outcomes behave as pathway properties. We therefore treat diagnostic safety and pathway integrity as the most plausible early gains from LHS implementation, while downstream outcome effects require the pragmatic evaluation designs specified in Section 7. These observations are presented as illustrative and hypothesis-generating, not as definitive causal estimates.

Box 1 **Implementation checklist: operating a rare-cancer measurement loop (SSN exemplar).**

***Core* *checklist* *(one* *page)***

**A. Set-up (one-time prerequisites)**

- Governance: CGB, MDSG, MDTB decision traceability, spoke quality leads.
- Minimum dataset and provenance; data quality gates; definition version control.

**B. Monthly cycle (run the system)**

- Data integrity gate: Missingness + plausibility + capture completeness.
- Dashboard: MDTB coverage/time, TTI, whoops rate; rapid-cycle actions.

**C. Quarterly cycle (compare and intervene)**

- Interpretability gate: Intent/histotype/complexity stratification; N/A rules; balancing indicators.
- Benchmark panel across Table 1 domains; approve interventions; check fidelity.

**D. Annual cycle (prove learning)**

- Outcomes report with follow-up adequacy; validity/equity/anti-gaming audit; annual learning report.

**E. Applicability rulebook (hard rules)**

- Use N/A explicitly (not silently dropped) when irrelevant, denominator unreliable, or program not deployed.

**F. Triggers and escalation**

- Amber: Sustained deterioration; root-cause review.
- Red: Safety-relevant deterioration; immediate governance action.

**G. Documentation standard**

- For each cycle: What changed, hypothesis, intervention owner, re-measurement + balancing indicators.

Abbreviations: CGB, Clinical Governance Board; MDSG, Measurement and Data Stewardship Group; MDTB, multidisciplinary tumor board; N/A, not applicable; SSN, Swiss Sarcoma Network; TTI, time-to-treatment initiation.

**Table 1:** Minimum viable measurement backbone (compact).

Domain	Minimum Indicators (Examples)	Denominator/Time Window	Applicability (N/A Rules) *	Case-Mix/Stratification Anchors
Diagnostic safety & timeliness	Time-to-treatment initiation (TTI); time-to-final diagnosis & diagnostic concordance	Newly diagnosed cohort; median/IQR; interval decomposition where feasible	N/A if benign/watchful waiting; report missing timestamps	Histotype; institution/workflow; referral source; grade/site
Diagnostic yield & pathway quality	RMST/biopsy ratio (suspected → confirmed sarcoma); diagnostic pathway interval decomposition	Consecutive suspected-tumor biopsies; standardized suspicion taxonomy	N/A if biopsies outside capture; disclose capture completeness	Spoke vs. hub; symptom logic; pathway segment; institution
Oncologic outcomes	Local recurrence (time-to-LR); metastatic dynamics (timing/tropism/burden)	Curative-intent cohort; competing risk preferred; follow-up adequacy explicit	N/A for benign/palliative intent; separate cohorts	Grade; margins; pathway type; planned vs. unplanned; histotype/stage
Harms & complications	30-day postoperative morbidity; wound complications after preop RT	Standardized complication definitions; fixed window	N/A if complication capture not standardized; report missingness	ASA-Score; bone vs. STS; complexity; RT regimen
Function, PROMs & PREMs	Longitudinal HRQoL completion + change; biopsy PREM/PROM (anxiety/pain/satisfaction)	Predefined timepoints; completion + change metrics	N/A if PROM/PREM program not deployed; must be declared	Baseline function; intent; treatment type; communication pathway
Process fidelity & governance	MDTB coverage + time-to-MDTB decision; whoops/unplanned excision rate	Eligible cases; defined decision points; rolling capture	N/A only if alternate governance model exists (explicitly described); whoops N/A if external surgery data missing	Referral region; pathway; diagnostic access; complexity
Costs & resource stewardship	Episode costs stratified by complexity; resource use vs. tariff separation	Defined episodes; report resource use + cost perspective	N/A if costing method not harmonized; disclose method	STS-SCS/BT-SCS; malignancy; institutional practice

Abbreviations: ASA, American Society of Anesthesiologists; BT, bone tumor; HRQoL, health-related quality of life; IQR, interquartile range; LR, local recurrence; MDTB, multidisciplinary tumor board; N/A, not applicable; PREM, patient-reported experience measure; PROM, patient-reported outcome measure; RMST, restricted mean survival time; RT, radiotherapy; SCS, Surgical Complexity Score; STS, soft tissue sarcoma; TTI, time-to-treatment initiation. * Cross-site benchmarking requires capture completeness disclosure; otherwise, indicator is N/A for benchmarking.

## 3. LHS Architecture: Closed-Loop Learning Cycles

Each step is necessary, and none is sufficient alone [19,21]. Capture without harmonization produces non-comparable data; benchmarking without learning yields dashboards; learning without implementation yields inconsequential publications; and implementation without re-measurement yields unverified change [7,22]. Accordingly, the operating model specifies (i) minimum viable data primitives and decision traceability, (ii) a measurement backbone with explicit validity gates, and (iii) governed implementation with re-measurement to confirm impact and detect unintended consequences.

**Figure 2:**
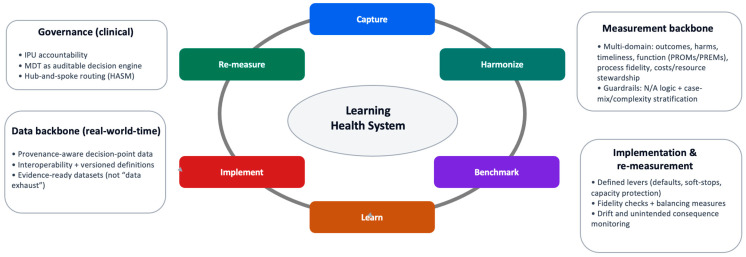
Closed-loop learning cycle for rare-cancer pathways. Minimal operating loop that converts routine care into auditable learning: capture of structured, time-stamped decisions at pathway interfaces; harmonization through version-controlled definitions; benchmarking with explicit denominators, applicability (“N/A”) rules, and stratification; learning via hypothesis-driven interpretation; implementation through governed workflow change; and re-measurement to confirm improvement and detect unintended consequences. Guardrails constrain interpretation and escalation when validity, safety, or equity signals deteriorate. How to read: Follow the arrows clockwise to trace how a pathway signal becomes an authorized intervention and is then re-evaluated under governance.

A rare-cancer Learning Health System (LHS) should be defined operationally: a governed clinical system that converts routine care into continuous improvement through measurement, feedback, and re-measurement [8,18,21,23]. The core requirement is not comprehensive data capture, but instrumentation of the decisions and pathway interfaces that determine outcomes and preventable harm, coupled with governance that renders those decisions traceable and comparable across sites. This operating logic is summarized as a closed-loop control cycle in Figure 2: capture → harmonize → benchmark → learn → implement → re-measure.

Operationally, “closed-loop” means that the system (i) captures structured data at the moment decisions are made (instrumented decision points), (ii) produces comparable indicators under explicit definitions and denominators (measurable outputs), and (iii) uses those outputs to authorize workflow change under governance and then re-measures impact. The unit of learning is therefore not a retrospective dataset but a time-stamped decision and its downstream consequences. This is the minimum structure required to make pathway interventions auditable and testable rather than anecdotal.

The LHS is operationalized as a closed-loop control cycle that converts routine care into auditable learning through instrumented decision points and measurable outputs (Figure 2). The loop is intentionally minimal: it specifies where data must be created (at decisions), how it must be rendered comparable (definitions), what must be produced (valid indicators), and how signals become change (governed implementation and re-measurement) [18,20,24]. Digital infrastructure is enabling but not determinative; whether learning is real depends on governance, definitional discipline, and implementation fidelity [10].

- Capture: Prospective, provenance-aware capture at decision points (triage, imaging/biopsy planning, MDTB intent, treatment exposure, key outcomes).
- Harmonize: Standardized definitions and a versioned data dictionary to preserve comparability across sites.
- Benchmark: Longitudinal indicator computation with explicit denominators, exclusions, applicability (“N/A”) rules, and required stratification.
- Learn: Hypothesis-driven interpretation using pragmatic evaluation designs; escalation to causal learning only when estimands and bias controls are explicit.
- Implement: Governed workflow change and network support (interventions are specified, owned, and fidelity-checked).
- Re-measure: Confirmation of improvement and detection of unintended consequences using balancing measures and drift monitoring.

Operationally, the loop links directly to the measurement backbone (Table 1; Supplementary Tables S1 and S2) and to the governance and implementation mechanisms that prevent “dashboard medicine” (Figure 2, Box 1, Appendix A).

Worked example (TTI): The network defines “time-to-treatment initiation” as MDTB decision date → start of definitive therapy, with exclusions (watchful waiting; planned neoadjuvant staging) and applicability rules (N/A if intent is palliative). Capture: MDTB decision date and therapy start are recorded prospectively. Harmonize: Definition/version is fixed across sites. Benchmark: TTI is reported stratified by histotype/intent/complexity, alongside capture completeness. Learn: If one spoke shows prolonged TTI, root-cause review distinguishes capacity constraints from referral delay or missing timestamps. Implement: A targeted intervention is approved (e.g., protected biopsy/imaging slots; routing rule; MDTB template default), with an owner and fidelity checks. Re-measure: TTI is re-evaluated with balancing measures (repeat biopsy, complication rate, diagnostic revisions) and escalation triggers (amber/red) if harm signals rise. This worked example corresponds to the TTI indicator in Table 1; the full operational definition (denominator/exclusions, applicability ‘N/A’ logic, and stratification anchors) is detailed in Supplementary Tables S1 and S2.

## 4. Minimum Viable Data Backbone and Definitions

A rare-cancer LHS fails if it cannot produce comparable, time-stamped, provenance-aware data at the points where decisions are made [25,26,27,28,29]. The minimum viable data backbone is intentionally smaller than “capture everything,” but sufficiently structured to support valid benchmarking, pathway mapping, and improvement [10]. Each core element should be defined, versioned, and traceable to source (provenance), so that longitudinal signals are interpretable and resistant to definitional drift.

Minimum data primitives (recommended):

- Patient baseline: Age, comorbidity proxies, baseline function where feasible.
- Tumor descriptors: Site, size, depth, grade, histotype; staging where appropriate.
- Pathway timestamps: First presentation/network entry, imaging order/completion, biopsy date/type, pathology sign-out, MDTB decision date, definitive treatment start.
- Treatment exposure fidelity: What was delivered (surgery type, margin status; radiotherapy (RT) schedule; systemic chemotherapy exposure; if applicable: interventional therapy, e.g., cryotherapy, radiofrequency ablation, etc.), not only what was planned.
- Outcomes: Local recurrence, metastasis, survival where relevant; complications and harms.
- Patient-centered signals: PROMs/PREMs via a feasible longitudinal strategy (completion rate and timing are part of the signal).

Definitional discipline is not optional. Benchmarking indicators (e.g., diagnostic yield/timeliness metrics, cost-by-complexity) require explicit taxonomies, denominators, and clear separation of resource use from pricing/tariffs; otherwise, comparisons are prone to artifact and are operationally non-actionable (Table 1; Supplementary Tables S1 and S2).

## 5. Measurement Backbone: What to Measure, How Often, and Why

The measurement backbone must satisfy three constraints simultaneously: validity (alignment with patient-relevant benefit and harm), feasibility (high completion with minimal burden), and actionability (a clear link to modifiable system levers). In rare cancers, where heterogeneity is high, and denominators are small, this requires explicit resistance to single-proxy “quality” claims and surrogate-driven escalation [30,31]. Accordingly, the measurement set should be multi-domain, spanning oncologic outcomes, harms/complications, diagnostic timeliness and pathway performance, function and PROMs/PREMs, process fidelity/governance, and cost/resource stewardship [25,27,32,33,34,35,36].

Two guardrails are non-negotiable. First, applicability logic (“N/A”) must be explicit to prevent invalid benchmarking when an indicator is clinically irrelevant to intent, not implemented, or not reliably captured. Second, case-mix and complexity stratification are required to prevent perverse incentives and false inference; aggregated comparisons without stratification are treated as non-interpretable for accountability.

Rare-cancer practice evolves, and uncontrolled evolution can invalidate longitudinal benchmarking. Therefore, the operating model treats indicator continuity as a governed process: definitions, denominators, and exclusions are version-controlled, and measurement changes require explicit documentation of what changed, why, and expected discontinuities. For major shifts (e.g., new diagnostic modalities, adoption of AI-based tools, or new treatment standards), the model recommends staged deployment with “shadow mode” and parallel reporting (legacy and updated indicator definitions co-reported for a defined period) to quantify discontinuities and prevent silent drift. Where comparability cannot be preserved, indicators are updated with explicit version boundaries rather than retrospectively blended.

Table 1 specifies a minimum viable indicator set suitable for real-world-time benchmarking, with operational definitions, denominators, exclusions, and applicability rules detailed in Supplementary Tables S1 and S2. Operational guardrails (including capture-completeness reporting, escalation triggers, and anti-gaming checks) are specified in Box 1 and Supplementary Tables S1 and S2. Indicators that are not applicable (“N/A”) are explicitly labeled to preserve validity.

Credible benchmarking across institutions requires explicit validity gates that address heterogeneity in capture. In this framework, an indicator is benchmarked only if (i) definitions, denominators, exclusions, and start/stop rules are standardized and version-controlled; (ii) capture completeness is reported (missingness and denominator integrity are treated as part of the signal, not suppressed); and (iii) interpretability constraints are satisfied through explicit applicability (“N/A”) rules and mandatory case-mix/complexity stratification. If any gate fails—e.g., external procedures, follow-up events, or complications are not captured consistently—cross-site comparison is prohibited, and the indicator must be declared N/A (or restricted to within-site learning) until capture is improved. These gates, drift audits, and anti-gaming safeguards are operationalized through the governance cadence (Box 1; Appendix A) and indicator-level failure mode mapping (Supplementary Tables S1 and S2).

## 6. From Measurement to Change: Implementation Under Governance

Measurement only generates value when it is explicitly linked to implementation authority, ownership, and re-measurement [6,37,38,39]. In a rare-cancer LHS, the dominant failure mode is not lack of data but lack of translation: signals remain descriptive, dashboards proliferate, and behavior does not change. Therefore, the operating model treats implementation as a governed step in the loop, not as an implicit consequence of transparency.

Implementation is anchored in workflow-level interventions rather than education alone [25,26]. High-leverage mechanisms include defaults and soft-stops in diagnostic sequencing, protected capacity for imaging and biopsy, standardized MDTB decision capture, and spoke-level support for referral routing. To prevent surrogate-driven optimization, implementation is conditioned on balancing measures and predefined escalation thresholds. Interventions that accelerate pathways (e.g., reducing time-to-treatment) must be paired with safety and fidelity checks (e.g., diagnostic yield/accuracy proxies, re-work rates such as repeat imaging/biopsy, complications, avoidable escalations, and patient-reported outcomes where feasible), and improvement is not accepted if gains in speed coincide with increased harm. These guardrails are specified in the implementation checklist and governance cadence (Box 1) and aligned to indicator-level failure mode mapping (Supplementary Tables S1 and S2). Crucially, interventions are specified ex ante, assigned an owner, and paired with explicit fidelity criteria and balancing indicators to detect unintended consequences [40].

Governance determines whether implementation is credible [41]. Decisions to intervene, escalate, or pause are taken within defined governance structures and cadence, ensuring that pathway change is coordinated across the network rather than localized or opportunistic. This prevents both under-reaction (signals ignored) and over-reaction (single metrics driving disproportionate change).

The practical logic is summarized in Box 1, which operationalizes implementation as a repeatable cycle: interpret signals under validity guardrails, authorize targeted interventions, verify execution, and re-measure impact. This closes the loop and distinguishes a learning system from retrospective audit or quality reporting.

A key implication of Box 1 is that a rare-cancer learning system is not an IT platform project, but a governed clinical operating model in which measurement validity, accountability, and implementation fidelity determine whether learning occurs. Digital infrastructure enables timely capture, harmonization, and benchmarking, yet it does not substitute for explicit governance of denominators, applicability (“N/A”) rules, case-mix stratification, and anti-gaming safeguards. Accordingly, the claims of this manuscript are intentionally framed within improvement science: the operating model is designed to generate auditable, reproducible pathway change and to support pragmatic evaluation (e.g., interrupted time series or stepped-wedge deployment) rather than to assert causal effects by default from observational dashboards.

## 7. Evaluation Designs and Risks (Bias, Drift, Unintended Consequences)

The operating model is designed to be testable without over-claiming causality [39,42]. Because many pathway-level interventions in rare cancers—such as referral routing, diagnostic sequencing, MDTB timing, or capacity protection—are not readily randomizable, evaluation must rely on pragmatic designs that preserve temporal ordering, denominators, and context. Accordingly, pragmatic designs such as interrupted time series, stepped-wedge implementation, and prospective real-world-time benchmarking are well-suited to assess system-level change [22].

To distinguish LHS-associated change from secular trends, interventions should be evaluated with explicit temporal structure and counterfactual logic. Interrupted time series analyses should use pre-specified intervention dates (e.g., rollout of routing rules, biopsy governance, protected capacity, or template-driven MDTB decisions) with segmented trend estimates, complemented by contemporaneous “control” indicators or cohorts not expected to change (negative controls) where feasible. When interventions are rolled out sequentially across sites or pathway components, stepped-wedge deployment provides additional separation from time effects. Across designs, stratification by intent/histotype/complexity and version-controlled indicator definitions are required to prevent apparent improvement driven by case-mix shifts or definitional drift rather than true pathway effects.

SSN’s published analyses motivating this blueprint have primarily used observational cohort designs (including adjusted comparisons of integrated versus fragmented pathways) to quantify associations with endpoints such as local recurrence. These studies are not presented here as definitive causal estimates for all pathway levers; rather, they motivate why formal evaluation designs are required. For prospective pathway interventions, the operating model prioritizes pragmatic designs with explicit counterfactual logic—interrupted time series with pre-specified intervention dates and segmented trends, stepped-wedge rollout across sites or pathway components, and contemporaneous control indicators/cohorts where feasible—combined with pre-specified estimands, version-controlled definitions, and capture-completeness disclosure to reduce attribution error from case-mix shifts and definitional drift.

Two classes of risk require explicit management [39,43,44]. First, attribution risk: observed improvements may reflect case-mix shifts, referral behavior changes, or definitional drift rather than true pathway effects. Concretely, the model requires reporting of capture completeness, referral-source distributions, and key case-mix anchors over time (e.g., histotype, intent, complexity strata) alongside indicator trends, and it mandates version boundaries when definitions change. These steps do not “prove” causality, but they reduce the probability that the apparent improvement is an artifact. This risk is mitigated—but not eliminated—through pre-specified estimands, explicit applicability (“N/A”) rules, complexity stratification, and versioned indicator definitions [40]. Second, surrogate drift: improvement in a single metric (e.g., shorter time-to-treatment) may be clinically neutral or harmful if achieved through misclassification, unnecessary interventions, or increased complications. For this reason, the operating model mandates multi-domain measurement and balancing indicators rather than reliance on isolated proxies.

Accordingly, the claims supported by this framework are intentionally bounded. The LHS operating model does not assert causal superiority of specific treatments or technologies. Instead, it supports credible improvement science: the ability to detect variation, implement pathway-level change under governance, and verify whether intended improvements occur without unintended harm [45]. More formal causal inference—including target trial emulation or model-assisted decision support—becomes appropriate only when data completeness, estimand clarity, and bias controls are explicitly satisfied. This boundary is intentional: pathway governance and measurement validity are prerequisites for credible evaluation, not substitutes for comparative effectiveness research. Therefore, the operating model does not assign causal credit to specific treatments or technologies; it specifies the conditions under which causal claims may later be evaluated with explicit estimands and bias controls.

By maintaining this discipline, the proposed LHS avoids both extremes: underpowered descriptive dashboards and overconfident causal claims. It provides a reproducible foundation for continuous learning in rare-cancer care while preserving scientific humility.

## 8. Discussion: Implications for Rare-Cancer Care, Measurement, and Evidence

### 8.1. Transferability and Prerequisites for Adoption

The operating model is intentionally generic, but not context-free. Transferability does not require reproducing the Swiss Sarcoma Network (SSN) as an institution; it requires a small set of prerequisites: explicit clinical governance, auditable multidisciplinary decisions, defined referral routing, and a data backbone that generates comparable observations with timestamps and provenance at key decision points [46,47]. Where these prerequisites are absent, attempts to implement Learning Health Systems risk devolving into fragmented quality reporting or isolated digital initiatives.

Here, we define “capacity to learn” operationally. A network can learn if it (i) records decisions with traceable timestamps, (ii) makes them comparable across sites through versioned definitions and denominators, and (iii) runs governed feedback cycles that authorize change and then re-measure its effects. This capacity is measurable (e.g., capture completeness, decision traceability, definition/version audits), even before any single intervention shows a proven downstream outcome effect.

To avoid siloed learning, networks must connect to external datasets (e.g., national cancer registries) rather than assuming their internal data is sufficient. In this operating model, interoperability rests on three minimal elements: version-controlled definitions, provenance-aware timestamps at decision points, and a linkage strategy for secure connection to external sources where legally permissible. Registries add what network datasets often miss—outcome completeness (e.g., vital status, long-term follow-up) and external reference distributions for case-mix calibration. The network backbone adds what registries often lack—granular pathway interfaces (routing, diagnostic sequencing, biopsy planning), auditable intent-setting in MDTs, and signals of implementation fidelity. The integration is therefore modular: linkage for completeness and context, and network instrumentation for change.

The model does not require full centralization. Hub-and-spoke preserves local delivery while ensuring early access to expertise and governed decisions [15,48]. This matters in rare cancers: over-centralization can create access barriers, whereas under-governance perpetuates preventable harm and makes pathways non-learnable. Therefore, transferability depends less on volume thresholds and more on pathway discipline and accountability.

### 8.2. Hybrid Evidence: Complementing, Not Replacing, Trials

A core contribution is to place real-world-time data within a hybrid evidence stack, not as a substitute for randomized trials [49]. Many determinants of rare-cancer outcomes—diagnostic delay, biopsy planning, MDT timing, and referral routing—are pathway interventions that conventional trial designs rarely address. The LHS operating model supports structured observation and pragmatic evaluation of these interventions under real-world conditions, using designs suited to system change [23].

The framework also enforces claims discipline. Benchmarking describes variation; it does not, by itself, justify causal inference. Escalation to causal learning (e.g., target trial emulation or model-assisted decision support) requires explicit estimands, bias-control strategies, and stable data generation processes [43,50]. By embedding these requirements upstream—in governance and measurement design—the LHS makes stronger inference possible without conflating description with causation.

### 8.3. Measurement Validity and Protection Against Gaming

Measurement is both the strength and the vulnerability of learning systems [38]. Without explicit guardrails, benchmarking can incentivize superficial optimization: selective capture, denominator manipulation, or escalation driven by surrogates rather than patient benefit. We therefore treat two protections as non-negotiable: applicability logic (“N/A”) and case-mix/complexity stratification. Together, they prevent invalid comparisons and reduce pressure to improve metrics by changing denominators rather than care.

Equally important is the insistence on multi-domain measurement [51,52,53]. No single indicator—timeliness, margin status, complication rate, or cost—can serve as a proxy for quality in rare cancers. Accordingly, we treat quality as a composite construct spanning outcomes, harms, function, process fidelity, and resource stewardship. This aligns measurement with patient benefit and system value rather than with easily optimized surrogates.

### 8.4. Value-Based Healthcare Without Managerial Reductionism

The operating model offers a concrete pathway toward value-based healthcare in rare cancers without reducing value to managerial abstractions [10,16,54]. By linking measurement to governed pathway change, costs and outcomes become interpretable in the context of intent and complexity. This enables a serious discussion of efficiency—what resources are used, for whom, and with what benefit—without confusing accounting artifacts with clinical performance.

Crucially, the model does not treat measurement as external control. Measurement sits inside clinical governance and supports collective learning rather than individual sanction. This distinction is central for clinician engagement and for sustaining improvement.

### 8.5. Roadmap to Advanced Learning: Necessary but Downstream

The operating model clarifies the relationship between foundational learning systems and more advanced ambitions, such as causal analytics or digital twins [10,30,46]. We do not reject advanced analytics; we sequence it. Without stable data generation, explicit governance, and validated measurement, advanced methods amplify noise and bias. With these foundations in place, they become legitimate extensions of the learning system rather than speculative overlays.

Our contribution is not to describe a final state, but to define minimum conditions under which learning in rare-cancer care is scientifically credible and operationally useful. The SSN exemplar shows that these conditions are achievable and that the benefit extends beyond single interventions to the system’s capacity to learn. While recent work often frames Learning Health Systems as a broad socio-technical concept for safe AI deployment, we take a practice-oriented stance: we specify the governance, measurement, and implementation primitives that make rare-cancer pathways learnable, with AI positioned as a downstream capability rather than the primary driver [55].

### 8.6. Limitations

This article is a perspective and implementation framework, not an effectiveness study. The model is grounded in SSN’s published trajectory and implementation logic, but its causal impact on outcomes (e.g., local recurrence, metastatic dynamics, costs) requires prospective evaluation with explicit estimands and bias control. Generalizability is context-dependent. SSN operates within specific regulatory, reimbursement, and data-infrastructure conditions. Transfer to other jurisdictions may require adapted governance arrangements, legal basis/consent models, and interoperability strategies. Measurement feasibility will vary across networks. This is particularly true for longitudinal PROMs/PREMs and for capture of external events (e.g., outside biopsies or unplanned excisions). The blueprint mitigates these risks through explicit applicability (“N/A”) rules, capture-completeness reporting, and stratification, but benchmarking remains sensitive to missingness and surveillance intensity. Finally, unintended consequences are plausible. Accelerating processes without balancing measures can increase misclassification, unnecessary procedures, or harm. The model addresses this through multi-domain measurement and escalation governance, but residual risk remains.

## 9. Conclusions: A Replicable Blueprint and Next Steps

Rare-cancer care will not become reliable or evidence-generating by incrementally optimizing isolated decisions. Outcomes and value are driven primarily by pathway integrity, governance, and the ability to learn from routine care. This perspective defines a pragmatic operating model for a rare-cancer Learning Health System and makes these drivers explicit and actionable.

Using the Swiss Sarcoma Network as an exemplar, we show how governed multidisciplinary decisions, hub-and-spoke routing, and a minimum viable real-world-time data backbone can be combined into a closed-loop learning cycle. By specifying ownership, applicability rules, stratification, and governance mechanisms for implementation, the model translates measurement into change while preserving claims discipline.

The contribution is not a platform, a metric set, or a single intervention. It is a transferable operating model for running rare-cancer care as a high-reliability learning system. Establishing this foundation is a prerequisite for credible benchmarking, accountable value assessment, and advanced capabilities such as causal analytics and digital twins.

## Data Availability

No new data were created or analyzed in this study. Data sharing is not applicable to this article.

## Supplementary Materials

The following supporting information can be downloaded at: https://www.mdpi.com/article/10.3390/cancers18040643/s1. Table S1: Expanded operational definitions and governance mapping; Table S2. Operationalization and safeguards (minimum viable).

## Abbreviations

The following abbreviations are used in this manuscript:

ASA	American Society of Anesthesiologists
BT	bone tumor
CGB	Clinical Governance Board
EHR	electronic health record
HASM	hub-and-spoke model
HRQoL	health-related quality of life
ICU	intensive care unit
IQR	interquartile range
KPI	key performance indicator
LHS	Learning Health System
LOS	length of stay
LR	local recurrence
MDSG	Measurement and Data Stewardship Group
MDTB	multidisciplinary tumor board
N/A	not applicable
OR	operating room
QI	Quality Indicator
PREM	patient-reported experience measure
PROM	patient-reported outcome measure
RMST	restricted mean survival time
RT	radiotherapy
SAE	serious adverse event
SCS	Surgical Complexity Score
SSN	Swiss Sarcoma Network
STS	soft tissue sarcoma
TTI	time-to-treatment initiation

## Appendix A

Measurement governance for a rare-cancer Learning Health System (SSN exemplar)

Purpose: Specify the minimal governance required to ensure that the measurement backbone produces valid, actionable, and non-gameable signals.

Structure and roles:

(1) Clinical Governance Board (CGB): Accountability owner; approves indicators, definitions, targets, and workflow changes.
(2) Measurement and Data Stewardship Group (MDSG): Semantic owner; maintains data dictionary and versioning; runs data quality and audits.
(3) Multidisciplinary tumor board (MDTB) conference: Decision engine; generates traceable intent and decision metadata.
(4) Spoke quality leads: Implementation owners at the edge; ensure capture fidelity and referral pathway improvement.

Cadence:

- Monthly: Operational dashboard (missingness, MDTB coverage/time, TTI, whoops) and rapid-cycle actions.
- Quarterly: Stratified benchmarking panel, outlier review, intervention approval, and fidelity.
- Annual: Outcomes and validity audit (definitions, equity, anti-gaming), annual learning report.

Rules:

- Version control for definitions; trend breaks documented.
- N/A rulebook used explicitly (never silent dropping).
- Case-mix and complexity stratification required for comparisons.
- Anti-gaming safeguards: Balancing indicators, denominator drift monitoring, capture completeness statements.
- Escalation triggers: Amber (sustained deterioration); red (safety-relevant deterioration).
