# Supplementary material for: From Network Governance to Real-World-Time Learning: A High-Reliability Operating Model for Rare Cancers
Source: Cancers (Basel). 2026 Feb 16;18(4):643. doi: 10.3390/cancers18040643 (PMC12939146; doi:10.3390/cancers18040643)
Supplement: Supplementary file 1 [file cancers-18-00643-s001.zip › cancers-4147217-supplementary.pdf]

**Supplementary Table S1. Expanded operational definitions and governance mapping**

Note: Table S1A specifies indicator logic (definition, denominator, exclusions, and explicit N/A applicability). Table S1B specifies operationalization (minimum data elements, cadence, ownership, and typical failure modes with balancing checks).

Abbreviations: BT, bone tumor; HRQoL, health-related quality of life; ICU, intensive care unit; IQR, interquartile range; LOS, length of stay; LR, local recurrence; MDTB, multidisciplinary tumor board; N/A, not applicable; OR, operating room; PREM, patient-reported experience measure; PROM, patient-reported outcome measure; RMST, restricted mean survival time; RT, radiotherapy; SAE, serious adverse event; SCS, ; STS, soft tissue sarcoma; TTI, time-to-treatment initiation.

| Domain | Indicator | Operational definition (minimum) | Denominator / exclusions and N/A rule |
|---|---|---|---|
| Diagnostic safety & timeliness | Time-to-treatment initiation (TTI) | Days from network entry/definitive suspicion to start of first definitive treatment (surgery/RT/systemic as intent defines/if applicable: interventional therapy); report median and IQR; define intent and start-point explicitly. | All newly treated cases entering the network in the period; exclude benign/watchful waiting; report missing timestamps. N/A: N/A if no treatment is intended (benign/active surveillance) or if start-point cannot be defined consistently. |
| Diagnostic safety & timeliness | Time-to-final diagnosis and diagnostic concordance | Working days biopsy to signed final diagnosis; concordance local vs reference pathology using pre-defined categories; report non-diagnostic biopsies separately. | All biopsied suspected sarcoma cases with available sign-out; exclude purely imaging-based benign entities. N/A: N/A if reference pathology pathway not implemented; then report time-to-diagnosis only and state governance model. |
| Diagnostic yield / pathway quality | RMST / biopsy ratio (suspected to confirmed sarcoma) | Ratio of confirmed malignant sarcoma among biopsies performed under a standardized 'suspicious mass' taxonomy; disclose capture completeness. | Consecutive biopsies for 'suspicious' tumors; exclude biopsies without final diagnosis; report denominator capture. N/A: N/A if biopsies are not captured across sites (denominator incomplete); must |

| | | | |
|---|---|---|---|
| | | | explicitly disclose and prioritize capture. |
| Diagnostic yield / pathway quality | Diagnostic interval decomposition | Decompose total diagnostic interval into sub-intervals (e.g., first presentation to imaging; imaging to biopsy; biopsy to MDTB; MDTB to treatment) to identify bottlenecks. | All suspected sarcoma referrals with required timestamps; symptom onset optional (high missingness expected). N/A: N/A if timestamps are not reliable/standardized; report missingness and implement timestamp capture first. |
| Oncologic outcomes | Local recurrence (time-to-LR) | Time to first LR after definitive local therapy; competing-risk estimation preferred; follow-up adequacy stated. | Curative-intent cohort with defined follow-up; exclude benign/palliative intent; define index therapy. N/A: N/A for benign or purely palliative intent; N/A if follow-up insufficient for interpretation (must state). |
| Oncologic outcomes | Metastatic dynamics (timing, tropism, burden) | Time to first metastasis; site distribution; burden categories; interpret with surveillance cadence caveats. | Cohort with defined follow-up and ascertainment; specify imaging cadence or adjust if feasible. N/A: N/A if metastatic ascertainment is unreliable due to heterogeneous imaging without documentation. |
| Harms & complications | 30-day postoperative morbidity | Any complication within 30 days, graded consistently (e.g., Clavien-Dindo); report rate and severity distribution. | All eligible resections in period; exclude minor procedures only if pre-defined; report missingness. N/A: N/A if complication capture/coding not standardized; implement capture before benchmarking. |
| Harms & complications | Wound complications after preoperative RT | Wound complication within a fixed post-op window after preop RT and surgery; consistent definitions across sites. | All preop RT plus surgery cases; define window and event types; report reconstruction context. N/A: N/A if RT exposure or wound |

| | | | |
|---|---|---|---|
| | | | follow-up window cannot be captured consistently. |
| Harms & complications (measurement validity) | SAE reporting adequacy | System-level completeness and transparency of severe adverse event reporting across protocols/regimens; used as a validity check rather than outcome benchmarking. | Trials or regimens assessed; stratify by therapy class where relevant. N/A: N/A for modalities without systemic therapy exposure; report separately. |
| Function, PROMs & PREMs | Longitudinal HRQoL completion and change | Completion rate at predefined timepoints plus change from baseline; report missingness and minimal clinically important change where feasible. | All eligible patients enrolled in PROM program with baseline; exclude those without baseline only if stated. N/A: N/A if PROM program not deployed at required timepoints; must be declared. |
| Function, PROMs & PREMs | Biopsy PREM/PROM (anxiety, pain, satisfaction) | Standardized peri-biopsy patient experience and symptom measures using fixed timing and anchors; interpret as an implementation quality signal. | Consecutive biopsy patients under standardized workflow; report timing adherence. N/A: N/A if not collected; state as gap and implementation target. |
| Process fidelity & governance | MDTB coverage and time-to-decision | Proportion of eligible cases discussed plus median time to MDTB decision; include decision traceability fields (intent, sequencing, exceptions). | All eligible suspected/confirmed sarcoma cases; define eligibility rules; report missingness. N/A: N/A only if an alternative governance model is used and explicitly described. |
| Process fidelity & governance | Unplanned resection ('whoops') rate | Proportion of new sarcoma cases referred after prior unplanned excision outside pathway; track over time as a pathway-failure marker. | All new sarcoma cases; requires capture of external surgeries and pathology; disclose completeness. N/A: N/A if external surgery data not accessible; must be declared and addressed. |
| Costs & resource stewardship | Episode cost/resource use | Resource use and costs for defined care episodes stratified by | Defined episodes with consistent costing method; specify |

| | | | |
|---|---|---|---|
| | stratified by complexity | surgical complexity (STS-SCS/BT-SCS); separate resource use from tariffs/prices. | perspective; report components. N/A: N/A if costing method not harmonized/comparable; disclose and report resource use only. |
| Costs & resource stewardship | Resource use vs tariff separation | Report resource utilization (LOS, OR time, ICU, implants) separately from tariffs/pricing; prevents price-as-cost confounding. | Same cohorts/episodes as cost analysis; ensure definitional consistency. N/A: N/A if resource-use measures unavailable; prioritize extraction before interpreting costs. |

**Supplementary Table S2. Operationalization and safeguards (minimum viable).**

Abbreviations: ASA, American Society of Anesthesiologists; CGB, Clinical Governance Board; EHR, electronic health record; HRQoL, health-related quality of life; ICU, intensive care unit; KPI, key performance indicator; LOS, length of stay; LR, local recurrence; MDTB, multidisciplinary tumor board; N/A, not applicable; OR, operating room; QI, ; RMST, restricted mean survival time; RT, radiotherapy; SAE, serious adverse event; SSN, Swiss Sarcoma Network; TTI, time-to-treatment initiation.

| **Indicator** | **Core data elements (minimum)** | **Frequency** | **Primary owner** | **Failure mode, safeguards, and typical lever** |
|---|---|---|---|---|
| TTI | Referral/network entry date; imaging and biopsy dates; MDTB date; treatment start; intent | Monthly/Quarterly | CGB + QI lead | Failure: definitional drift or selective exclusions reduce apparent TTI. Safeguard: version control + missingness monitoring; balancing check: misdiagnosis and complication signals; lever: protected slots and EHR defaults. |
| Time-to-final diagnosis & concordance | Biopsy date/type; pathology sign-out; reference review and concordance category | Quarterly | Pathology lead + QI | Failure: faster sign-out at the expense of misclassification. Safeguard: concordance tracking; lever: standardized biopsy planning and expert review. |

| | | | | |
|---|---|---|---|---|
| RMST / biopsy ratio | Suspicion taxonomy; biopsy counts; final diagnosis; benign/malignant classification | Quarterly | Diagnostic pathway lead | Failure: gaming by narrowing the 'suspicious' label or denominator loss. Safeguard: audit taxonomy use and capture completeness; lever: triage algorithms and referrer pathways. |
| Diagnostic interval decomposition | Key timestamps across pathway segments; optional symptom onset | Annual | QI lead + spoke leads | Failure: missing timestamps hide delays; recall bias for symptom onset. Safeguard: timestamp completeness KPI; lever: workflow redesign and hub responsiveness. |
| Local recurrence (time-to-LR) | Index therapy date; margin; RT; LR date; follow-up | Annual | Outcomes lead | Failure: loss-to-follow-up and heterogeneous surveillance. Safeguard: follow-up adequacy reporting; lever: whoops reduction, margin planning, MDTB sequencing. |
| Metastatic dynamics | Imaging dates; metastatic sites; burden categories; follow-up | Annual | Outcomes lead | Failure: detection bias from heterogeneous imaging intensity. Safeguard: document cadence; stratify by protocol; lever: risk-adapted surveillance. |
| 30-day morbidity | Surgery type; ASA-Score; tumor type; complications with severity grading | Quarterly/Annual | Surgical quality lead | Failure: under-reporting or inconsistent grading. Safeguard: standardized definitions; lever: perioperative protocols and reconstruction planning. |
| Wound complication | RT regimen; surgery date; wound event | Annual | RT + surgery co-leads | Failure: confounding by selection into RT regimen. Safeguard: |

| | | | | |
|---|---|---|---|---|
| s after preop RT | window; reconstruction details | | | stratify by site/regimen/complexity; lever: regimen selection and perioperative optimization. |
| SAE reporting adequacy | Therapy class; SAE completeness fields; reporting framework | Annual | Therapy lead + QI | Failure: false reassurance from incomplete reporting. Safeguard: completeness audit; lever: standard SAE capture and transparent reporting. |
| HRQoL completion + change | PROM instrument; baseline and follow-up timepoints; missingness flags | Quarterly | PROM lead | Failure: selective non-response masks worse outcomes. Safeguard: completion-rate targets; lever: embed capture in routine visits and automate. |
| Biopsy PREM/PROM | PREM/PROM items; timing; analgesia; communication metadata | Quarterly | Diagnostic pathway lead | Failure: inconsistent timing and reporting bias. Safeguard: standard timing windows; lever: patient briefing scripts and pain protocols. |
| MDTB coverage + time-to-decision | Eligibility logic; MDTB date; structured decision fields (intent/sequence/exceptions) | Monthly/Quarterly | MDTB chair + QI | Failure: 'paper compliance' without substantive decisions. Safeguard: decision traceability; lever: scheduling, triage, and templates. |
| Whoops rate | External surgery flag; pathology; referral region/source; timestamps | Quarterly | Network governance + spokes | Failure: under-capture of outside events or denominator manipulation. Safeguard: capture completeness statements; lever: soft-stops and referral pathways. |

| | | | | |
|---|---|---|---|---|
| Episode cost/resource use by complexity | Episode definition; utilization measures; cost components; complexity score | Annual | Finance + clinical co-lead | Failure: price-as-cost confusion and non-comparable accounting. Safeguard: separate utilization from pricing; lever: process standardization and predictive planning. |
| Resource use vs tariff separation | LOS; OR time; ICU; implants; tariffs/prices | Annual | Finance lead | Failure: apparent 'savings' driven by accounting shifts. Safeguard: report utilization separately; lever: reduce utilization drivers of cost. |
